# FlowCell-enriched circulating tumor cells as a predictor of lung cancer metastasis

**DOI:** 10.1007/s13577-021-00500-8

**Published:** 2021-02-12

**Authors:** Yan Lu, Yushuang Zheng, Yuhong Wang, Dongmei Gu, Jun Zhang, Fang Liu, Kai Chen, Lingchuan Guo

**Affiliations:** 1 Department of Pathology, The First Affiliated Hospital of Soochow University, Suzhou, 215000 Jiangsu China; 2 Department of Oncology, The First Affiliated Hospital of Soochow University, Suzhou, 215000 Jiangsu China; 3 Suzhou Centre for Disease Control and Prevention, Suzhou, 215007 Jiangsu China

**Keywords:** Circulating tumor cells, Lung cancer, Metastasis, Biomarker, 2-NBDG

## Abstract

Lung cancer is the most fetal malignancy due to the high rate of metastasis and recurrence after treatment. A considerable number of patients with early-stage lung cancer relapse due to overlooked distant metastasis. Circulating tumor cells (CTCs) are tumor cells in blood circulation that originated from primary or metastatic sites, and it has been shown that CTCs are critical for metastasis and prognosis in various type of cancers. Here, we employed novel method to capture, isolate and classify CTC with FlowCell system and analyzed the CTCs from a cohort of 302 individuals. Our results illustrated that FlowCell-enriched CTCs effectively differentiated benign and malignant lung tumor and the total CTC counts increased as the tumor developed. More importantly, we showed that CTCs displayed superior sensitivity and specificity to predict lung cancer metastasis in comparison to conventional circulating biomarkers. Taken together, our data suggested CTCs can be used to assist the diagnosis of lung cancer as well as predict lung cancer metastasis. These findings provide an alternative means to screen early-stage metastasis.

**Supplementary Information:**

The online version contains supplementary material available at 10.1007/s13577-021-00500-8.

## Introduction

It has been estimated that over 14,000 died of lung cancer in 2019 in the United States alone and one out of four cancer-related death are from lung cancer ranking the first regardless of gender [1]. Despite advances in treatment strategies, especially targeted therapies and immune therapies, prognosis remains poor due to widely metastatic tumors in the majority of patients at the time of diagnosis [2]. A considerable number of patients with early-stage lung cancer relapse due to unnoticed distant metastasis [3]. Hence, it is of special significance to capture the early event of metastasis and continuously monitor cancer progression to amend therapeutic strategy accordingly. Liquid biopsy-based biomarkers provide promising opportunities for the early detection of metastasis as well as monitor of cancer progression [4].

Circulating tumor cells (CTC), derived from the primary tumors into the circulation system, are considered as the “seeds” for metastasis [5], have recently gained substantial attentions as biomarkers for lung cancer diagnosis and treatment [6]. Isolated CTCs have been proposed to predict therapy responses and recurrences of lung cancers [7, 8]. Multiple methods for CTC isolation and detection have been established based on the detection of epithelial cell adhesion molecule (EpCAM)-positive and CD45-negative cells [6]. However, this approach neglected a large proportion of CTCs without EpCAM expression, especially when EpCAM is downregulated during epithelial-to-mesenchymal transition [9, 10]. Establishment of a novel method that isolates and characterizes bona fide CTCs will benefit cancer patients in many aspects. Recently, it has been shown that measuring glucose uptake with fluorescent-labeled glucose analog 2-[*N*-(7-Nitrobenz-2-oxa-1,3-diazol-4-yl)amino]-2-deoxy-d-glucose (2-NBDG) is a powerful alternative to identify CTC from the blood [11]. Additionally, staining of cellular markers facilitated further classification of CTCs captured [12].

Based on previous discoveries, we employed a novel method to capture, isolate and classify CTC with FlowCell system and analyzed the CTCs from a cohort of 302 lung cancer patients who went through segmentectomy or lobectomy. We isolated and classified the CTCs from these patients and analyzed the CTC counts from patients with various cancer subtypes as well as stages. Most importantly, the relationships between CTCs and metastasis were analyzed. With this enhanced method to capture, isolate and classify CTC, our data enriched the application of CTCs to assist lung cancer diagnosis and offered a promising strategy to predict the metastasis of lung cancer.

## Materials and methods

### Patients and study design

Overall, 302 patients were enrolled in this study at Department of Thoracic Surgery, the First Affiliated Hospital of Soochow University between April 2018 and December 2018. A compete record of clinical parameters, pathological and radiographic data were retrieved from the prospective database of the hospital (supplementary file_1). Distant metastasis was assessed according the radiographic data. Primary tumor, metastasis sites and tumor size were carefully assessed by experienced radiologists. The study was approved by the Ethics Committee of the First Affiliated Hospital of Soochow University. The approval number of Ethical Committee is 2020-161. The study was performed in accordance with the principles of Declaration of Helsinki.

### Samples collection and CTCs enrichment

Two tubes of 7.5 ml blood samples were drawn from peripheral vein and collected into 10 ml BD Vacutainer^®^ EDTA tubes (Becton, Dickinson and Company, NJ, USA). Upon collection, the tubes were flipped upside down for several times to fully mix the samples with anticoagulants. Briefly, blood samples were centrifuged and then the plasma layer were discarded. The rest were subjected to erythrocyte lysis. After centrifugation, nucleated cells were re-suspended in the PBS plus with 1% BSA and placed on the FlowCell^®^ CTC enrichment system (Polaris Biology, Shanghai, China) to eliminate the majority of both residual red blood cells (RBC) and white blood cells (WBC). Next, the enriched cells were incubated with Cy5 conjugated anti-CD45 antibody at 37 °C for 40 min in a culture medium containing RPMI1640 medium without glucose. Subsequently, all cells were exposed to 0.4 mM 2-Deoxy-2-[(7-nitro-2,1,3-benzoxadiazol-4-yl)amino]-d-glucose (2-NBDG) and hoechst33342 at a concentration of 0.5 g/ml for additional 20 min. After extensive rinsing with cold PBS, all cells centrifuged onto the glass were scanned and imaged by a high-content screening system, EVOS FL Auto 2 (Invitrogen, MA, USA) in three fluorescent colors (DAPI: nuclei, 2-NBDG: CTCs, CY5: CD45) as well as the bright filed. A computational algorithm analyzed the images and identified candidate tumor cells that were viable and exhibited high glucose uptake, followed by reviewing by experienced technicians. Based on fluorescence staining, high glucose uptake cells with high 2-NBDG intensity beyond the threshold and negative CD45 staining were recognized as potential CTCs.

### Classification of CTCs

The phenotypes of CTCs could emerge as indicators for epithelial mesenchymal transition (EMT) process. The classification was based on the markers expressed on the CTCs, including pan-CK and vimentin. Briefly, the cells were stained with 2-NBDG and CD45 as described above for CTCs detection. Subsequently, cells were fixed with 4% paraformaldehyde and incubated with primary antibody pan-CK Alexa Fluor 488 conjugate (Abcam, MA, USA), vimentin Alexa Fluor 594 conjugate and CD45 PerCP-Cy5.5 conjugate (Cell signaling technology, CA, USA) at 37 °C for 1 h and visualized under EVOS FL Auto 2 (Invitrogen, MA, USA). Only cells displayed both 2-NBDG and Alexa 488 were regarded as epithelial CTCs, 2-NBDG and Alexa 594 for mesenchymal CTCs, and 2-NBDG, Alexa 488 and Alexa 594 for mixed CTCs. All types of CTCs were CD45-negative.

### Statistical analysis

For comparison of multiple groups of data, one-way ANOVA analysis was performed. Two-sided p values less than 0.05 were considered statistically significant. ROC analysis was performed to compare the specificity and sensitivity of CTC numbers and serum biomarkers. The statistical analysis was done using Graph Pad Prism 7.0 (Graph Pad Software Inc., La Jolla, CA, USA).

## Results

### Isolation and classification of CTCs in lung cancer patients

**Fig. 1:**
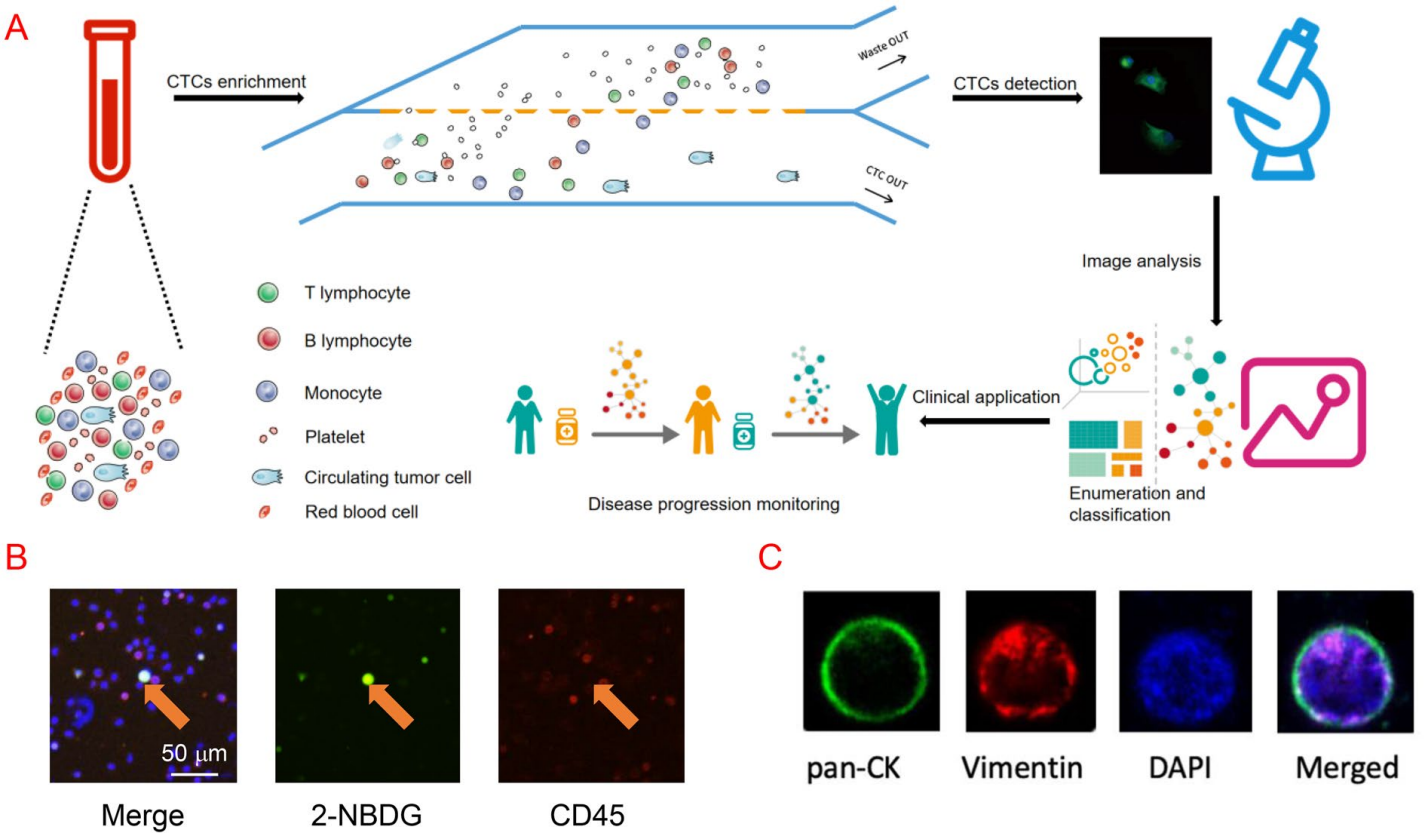
Metabolic based isolation and classification of CTC. **a** A schematic diagram of CTCs enrichment and detection procedure. **b** Typical staining images of CTCs. **c** Typical staining markers of CTCs

To isolate the CTCs in lung cancer patients, we employed the FlowCell CTCs enrichment system that separates CTCs in the circulation with a microfluidic-based particle separation technology. The FlowCell CTCs enrichment system utilizes a filter membrane with poles that are 12.5 μm in length and 7.3 μm in width. CTCs are retained by this membrane while allowing blood cells to pass through (Fig. 1a). To further validate that the cells isolated by FlowCell CTCs enrichment system are bona fide CTCs, we further stained the cell with 2-NBDG, cytokeratin, vimentin and CD45. Cells that are positive for 2-NBDG and negative for CD45 are classified as CTCs (Fig. 1b). Cells positive for cytokeratin and vimentin are classified as epithelial type CTCs and mesenchymal type CTCs, respectively. Cells that are cytokeratin and vimentin-double positive are classified as mixed type CTCs (Fig. 1c). By these criteria, the CTCs from 302 individuals that went through segmentectomy or lobectomy were isolated, classified and analyzed. Among these individuals, 255 patients were diagnosed as adenocarcinoma (AC), 19 patients were diagnosed squamous carcinoma (SCC) while the other 28 individuals carried benign tumor based on the pathological analysis.

### CTCs significantly increased as the tumor developed

**Fig. 2:**
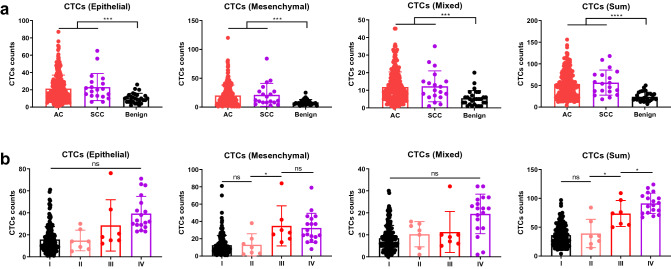
The comparison of CTCs counts of different CTCs types in various histological groups and disease stages. **a** CTCs counts in different histological groups. **b** CTCs counts in patients at different stages

We first analyzed the CTC counts based the pathological type of the patients and found that the CTC counts of patients diagnosed with lung cancer are significantly higher than that of individuals carrying benign tumor across all types of CTCs (Fig. 2a). We next categorized the patients by their staging and found that the CTC counts from patients at stage I and stage II are similar but the counts of mesenchymal type CTC and all CTC counts (Sum type) from patients at stage III are significantly higher than that from patients at stage II (Fig. 2b). Additionally, all CTC counts differentiate patients at stage III and patients at stage IV (Fig. 2b). Taken together, our results indicated that FlowCell-enriched CTCs effectively differentiated benign and malignant lung tumor and the total CTC counts increased as the tumor developed. Our data suggested FlowCell-enriched CTCs can assist diagnosis of lung cancer.

### CTCs serves as a promising predictor for lung cancer metastasis

**Fig. 3:**
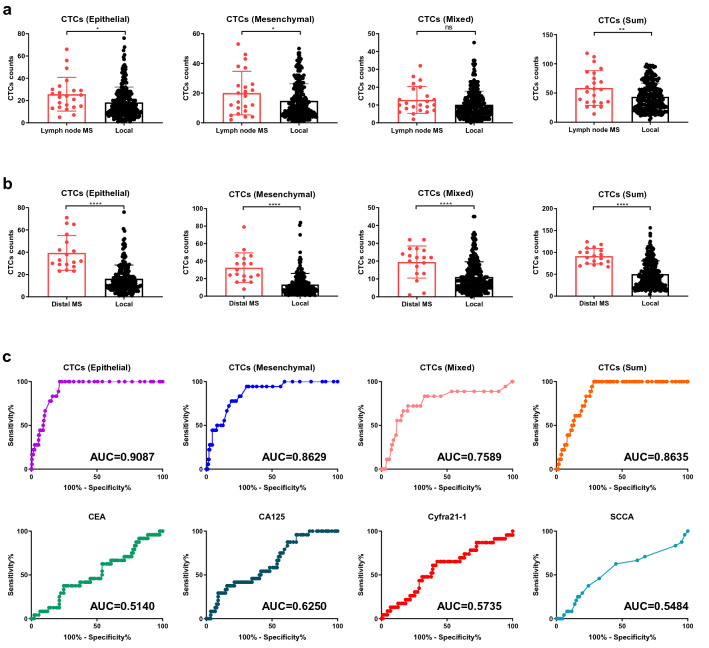
Elevated CTCs in patients with lymph node or distal metastatic sites. **a** CTCs counts in patients with (lymph node MS) or without (local) lymph node metastasis. **b** CTCs counts in patients with (distal MS) or without (local) distant metastasis. **c** ROC analysis of CTCs counts and serum biomarkers for evaluating metastasis risk

Since CTCs have long been considered as the seeds of cancer metastasis, we next evaluated the relationship between CTC counts and metastasis of lung cancer. We first analyzed the CTC counts from patients that have lymph node metastasis site or distal metastasis site and patients only have local tumor. The counts of epithelial type and mesenchymal type as well as the total number of CTCs are significantly higher in patients that have lymph node metastasis sites (Fig. 3a). The CTC counts from patients that have distal metastasis site were significantly higher than that from patients that only have local tumor regardless of CTC types (Fig. 3b). Taken together, our data suggested that monitoring the number of CTCs is an effective strategy to predict metastasis of lung cancer. Hence, we next plotted the receiver operating characteristic curve (ROC) with different types of CTC counts as well as well established circulating markers to assess the potential of CTC counts as predictors for lung cancer metastasis. The calculated area under the curve (AUC) for epithelial, mesenchymal, mixed and sum type CTCs were 0.9087, 0.8629, 0.7589 and 0.8635, respectively (Fig. 3c). In comparison to the AUC calculated for the conventional circulating biomarkers, CTCs showed superior sensitivity and specificity to predict lung cancer metastasis. Hence, our data indicated that CTCs serve as a promising predictor for lung cancer metastasis (Table 1).

**Table 1:** Basline demographic and clinicopathological characteristics

Characteristics Patients (*n*)			
Total evaluable patients 302			
Histological type	Adenocarcinoma	Squamous	Benign
Age (years)			
Median	60	67	56
Range	25–84	62–78	36–76
Gender			
Female	149	3	14
Male	106	16	14
TNM Stage			
I	193	2	NA
II	4	2	NA
III	7	0	NA
IV	39	5	NA
ND	12	10	NA

*ND* not determined, *NA *not appliacable

## Discussion

Hence, isolation and identification of bona fide CTC are crucial for clinical applications. Rapid advancements for isolation and characterization of CTC reflect the urgencies and necessities for application of CTC in clinics [13]. Several crucial limitations of the CellSearchs system, the only Food and Drug Administration (FDA) approved commercial system for CTC detection, drove the developments of CTC detection systems that aim at higher recovery rate and purity [13]. Here, we employed an enhanced CTCs enrichment method that separates CTCs by a microfluidic device based on crossflow filtration technology, distinguishes CTCs by measuring glucose uptake of fluorescent-labeled 2-NBDG and classify CTCs by cytokeratin and vimentin staining. In comparison to a recent report using CellSearchs system [14], FlowCell system showed a higher recovery rate of CTCs from lung cancer patients (median number 45 vs. 33) suggesting there may be more margin for setting up the threshold for the application of CTC in the diagnosis of lung cancer. However, we still observed in a considerable number of cancer patients that the CTC counts are in a smilar range to that from patients with benign tumors (Fig. 2). Hence, the cutoff value for lung cancer diagnosis requires larger sample sizes and more accurate pathological information. Moreover, 2-NBDG staining characterized the metabolically active cells among the cells in the blood but it is not reasonable to claim all 2-NBDG-positive cells are cancer cells. Meanwhile, whether CD45-positive cancer cells exist remains further exploration. Hence, our method is so far well-suited and accurate for the identification of CTC. Further fundamental study of the properties of CTC will improve the accuracy and efficiency of CTC isolation and identification.

It has been proposed that CTCs are promising biomarkers to predict metastasis in multiple types of cancers [15]. Also, classification of the CTCs isolated provides more accurate information for the predicition of metastasis [16–18]. Through the FlowCell system, we have successfully isolated and classified the CTCs. Our data illustrated that the counts of CTC are significantly higher in patients with metastasis. For patients with distal metastatic sites, the median number of epithelial type CTC counts is 32 while the median number of epithelial type CTC counts in the patients with only local tumor is 13 (Fig. 3b). This 2.4-fold change may be clinically relavent to predict distal metastasis and assist clinical examinations and treatment.

A cohort consisting 25 patients revealed that the changes in CTC counts were predictive of survival in patients with metastatic lung cancer receiving chemotherapy [19]. This study included samples from multiple timepoints, which suggested continuous monitoring the number of tumor cells in the circulation may serve as an additional index to predict survival as well as to guide clinical practice. We did not monitor CTCs in the lung cancer patients due to logistic reason, it would be of special significance to capture these data in future studies.

Cheng et al. [20] revealed that in lung cancer patients, higher positivity of CTCs was observed in the bone metastasis group than in the non-metastasis group. Most of the metastatic sites we observed in the present study are pleura pulmonalis. Futher analysis of other distal metastatic sites will gain more insight into the relationship between CTC and metastasis. Nonetheless, our data suggested that the counts of CTCs are a better predictor of metastasis than conventional circulating biomarkers, such as CA125 or CEA. Taken together, our method provided an alternative to screen for early-stage metastasis in a convenient and fast fashion.

In conclusion, our analysis of CTC counts in this cohort of 302 patients suggested that FlowCell system isolated CTCs assists the diagnosis of lung cancer and more importantly, serves as a biomarker to predict metastasis.

## Supplementary Information

Below is the link to the electronic supplementary material.

Supplementary file1 (XLSX 31 KB)
